# The Efficacy of 2‐Week Holter Monitoring for Detecting Atrial Tachyarrhythmia Recurrence After Initial Ablation in Patients With Atrial Fibrillation

**DOI:** 10.1002/joa3.70196

**Published:** 2025-09-21

**Authors:** Hirokazu Naganawa, Yuichiro Sakamoto, Yuko Uemura, Ryo Yamaguchi, Daisuke Yoshimoto, Maria Kristina Recio, Takahiko Suzuki

**Affiliations:** ^1^ Department of Cardiovascular Medicine Toyohashi Heart Center Toyohashi Japan

**Keywords:** atrial fibrillation, catheter ablation, Holter monitoring

## Abstract

**Background:**

Holter monitoring is widely used to detect atrial tachyarrhythmia (AT) recurrence after catheter ablation (CA) in patients with atrial fibrillation (AF). However, patients experience few subjective symptoms after CA, leading to potential underdiagnosis of recurrence. Two‐week Holter monitoring may be more effective compared to 24‐h Holter monitoring. This study aimed to evaluate the efficacy of 2‐week Holter monitoring for the detection of AT recurrence.

**Methods:**

From January 2019 to December 2021, 755 consecutive patients with AF (paroxysmal: 449, persistent: 256, long‐standing: 50) who underwent initial CA with wide‐area pulmonary vein isolation at our center were enrolled. Two‐week Holter monitoring was conducted at 3, 6, 12, 18, and 24 months after CA. Twenty‐four‐hour Holter monitoring was substituted for the first 24 h of the 2‐week Holter monitoring. Freedom from AT recurrence was defined as the absence of AT lasting > 30 s beyond a 3‐month blanking period.

**Results:**

Sixty‐nine (9.1%) patients dropped out. Among the remaining 686 patients, AT recurrence was detected over the 2‐year follow‐up period in 173 cases (25.2%) using 2‐week Holter monitoring and in 46 cases (6.7%) using 24‐h Holter monitoring (*p* < 0.001). Holter monitoring‐based analysis revealed that asymptomatic recurrence was significantly more common in patients with persistent and long‐standing AF (85.0%, 96/113 records) compared to those with paroxysmal AF (50.0%, 76/152 records) (*p* < 0.001).

**Conclusion:**

Two‐week Holter monitoring was significantly more effective than conventional 24‐h Holter monitoring for detecting AT recurrence after CA, particularly in patients with persistent and long‐standing AF.

## Graphical abstract

Two‐week Holter monitoring improves the detection rate and reduces underdiagnosis of atrial tachyarrhythmia recurrence after AF ablation, compared to 24‐h Holter monitoring.

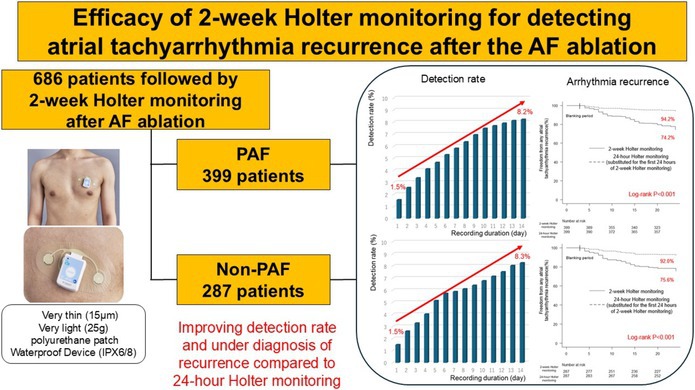

## 1 Introduction

Atrial fibrillation (AF) is the most common cardiac arrhythmia encountered in clinical practice and a significant risk factor for cardiovascular events, ischemic stroke, dementia, and mortality [1, 2, 3, 4, 5]. It also significantly reduces patients' quality of life, and its prevalence increases with advancing age [5, 6, 7]. Catheter ablation (CA) is an effective and widely accepted standard therapy for patients with AF that is recommended by many clinical guidelines [5, 8, 9, 10].

Holter monitoring is commonly used to detect atrial tachyarrhythmia (AT) recurrence and to evaluate long‐term outcomes after CA in patients with AF. However, patients often experience few subjective symptoms following CA, which may lead to underdiagnosis of recurrence [11]. Long‐term Holter monitoring has become the preferred method for follow‐up after CA with AF and is frequently employed in clinical trials [12, 13]. Compared to 24‐h Holter monitoring, 2‐week Holter monitoring may offer superior sensitivity in detecting AT recurrence.

Despite its potential advantages, there is a paucity of current literature discussing strict follow‐up using 2‐week Holter monitoring after CA in patients with AF. Therefore, this study aimed to evaluate the efficacy of 2‐week Holter monitoring for the detection of AT recurrence.

## 2 Methods

### 2.1 Patient Population

This was a single‐center, retrospective, observational study. We enrolled all patients with AF who underwent an initial radiofrequency catheter ablation (RFCA) with wide‐area pulmonary vein (PV) isolation using the CARTO system (Biosense Webster, Diamond Bar, CA, USA) at our hospital between January 2019 and December 2021. Patients who were not followed up with 2‐week Holter monitoring at the first post‐procedure follow‐up or who had an implanted device such as a pacemaker, implantable cardioverter‐defibrillator, or cardiac resynchronization therapy were excluded.

AF was defined as follows: paroxysmal AF (PAF) that terminated spontaneously or with antiarrhythmic drugs within 7 days, persistent AF (PEAF) that lasted > 7 days, and long‐standing AF (LSAF) that lasted > 1 year. All procedures were performed in accordance with the ethical standards of the institutional and/or national research committee and the 1964 Declaration of Helsinki and its later amendments or comparable ethical standards. This study was approved by the Institutional Review Board of Toyohashi Heart Center. Informed consent was obtained from all participants.

### 2.2 Ablation Procedure

Oral anticoagulation was administered for at least 1 month before the procedure and was continued throughout. Antiarrhythmic drugs were discontinued for at least five half‐lives before the procedure. Computed tomography and transesophageal echocardiography were performed to exclude left atrial (LA) thrombus and to visualize the LA, PV, and coronary sinus anatomy. The ablation procedure was guided by a 3‐dimensional mapping system (CARTO system; Biosense Webster, Diamond Bar, CA, USA) and performed using a 3.5‐mm irrigated tip catheter (ThermoCool SmartTouch SF catheter, Biosense Webster). All procedures were performed under deep sedation using fentanyl and propofol. PV isolation with wide‐area PV encircling was performed using RFCA guided by ablation index (AI) values (mainly > 550 at the anterior wall, > 400 at the posterior wall with an inter‐lesion distance < 6 mm) [14]. An esophageal temperature probe was used, and RF application was immediately interrupted if the luminal temperature reached 39.5°C, regardless of AI. In cases where posterior ablation lines were adjacent, additional RF applications were performed to close the posterior wall (“touching rings”). Cardioversion was performed to restore sinus rhythm if AF persisted after PV isolation. Atrial tachycardia mapping and ablation were performed if the AF was converted to atrial tachycardia during the procedure. After PV isolation, atrial burst pacing was performed; cardioversion was administered if AF was induced, and atrial tachycardia mapping and ablation were performed if atrial tachycardia was induced. Cavotricuspid isthmus ablation was performed in cases in which common atrial flutter was observed clinically or induced during the procedure. Additional ablations, such as roof line, bottom line, posterior wall isolation, or premature atrial contraction (PAC) ablation without AF triggers, were performed at the operators' discretion. Intravenous isoproterenol and adenosine triphosphate were administered to identify non‐PV foci and dormant conduction of isolated PVs; if they appeared, mapping and ablation were performed.

### 2.3 Follow‐Up

**FIGURE 1:**
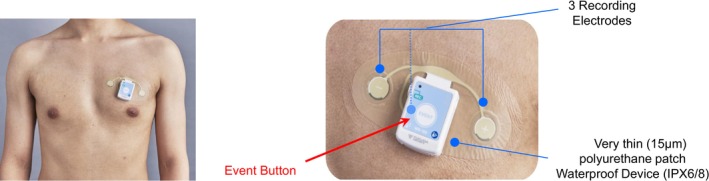
eMEMO WR‐100 (Fukuda Denshi, Tokyo, Japan). A 2‐week, leadless, adhesive patch electrocardiographic monitor. This device is extremely thin (15 μm), lightweight (25 g), and waterproof (IPX6). It features three recording electrodes and an event button at the center of the recorder. The monitor can continuously record a 2‐lead ECG signal for up to 1 week or a 1‐lead ECG signal for up to 2 weeks. ECG, electrocardiogram; IP, international protection.

Clinical evaluation and 12‐lead electrocardiography (ECG) were conducted at 1, 3, 6, 12, 18, and 24 months after the procedure, or in cases of arrhythmic symptoms. Two‐week Holter monitoring (WR‐100; Fukuda Denshi, Tokyo, Japan) was conducted 3, 6, 12, 18, and 24 months after the procedure. The device is shown in Figure 1. Twenty‐four‐hour Holter monitoring was substituted for the first 24 h of 2‐week Holter monitoring, ensuring that both groups were followed up using the same device. Patients wore the device for 2 weeks whenever possible; however, if this was not feasible due to holidays or work commitments, usage for more than 10 days was considered acceptable. If the device detached within 10 days of attachment, the patient was asked to visit the hospital for reattachment whenever possible. This device could detect arrhythmia semiautomatically, and the final data were manually reviewed to confirm the arrhythmia. The whole 2‐week Holter records were initially reviewed by the clinical technologist to identify arrhythmic events. These identified events were finally confirmed by the physician.

Antiarrhythmic drugs were discontinued in all the patients immediately after the procedure. During the acute phase, if AF recurred due to inflammation or other factors that caused the symptoms, antiarrhythmic drugs were administered; however, these drugs were consistently discontinued within 2 months of the procedure. All patients received anticoagulation therapy for 3 months after CA. Patients with a CHADS2 score of 0 or 1 without recurrence were considered for the discontinuation of anticoagulation therapy between 3 and 6 months after CA.

### 2.4 Outcome

The primary outcome of this study was freedom from any AT recurrence, defined as the absence of any AT lasting > 30 s beyond the 3‐month blanking period during the 2‐year follow‐up period. We investigated the comparison of recurrence detection rates in the 2‐week and 24‐h Holter monitoring. Additionally, patients with AF were divided into paroxysmal AF (PAF) and non‐paroxysmal AF (PEAF and LSAF) (NPAF) groups to analyze asymptomatic recurrence rates.

### 2.5 Statistical Analysis

All statistical analyses were performed using EZR (Saitama Medical Center, Jichi Medical University, Saitama, Japan), which is a graphical user interface for R (The R Foundation for Statistical Computing, Vienna, Austria) [15]. Continuous variables were expressed as means and standard deviations. For continuous data, groups of normally distributed and homoscedastic variables were compared using Student's *t*‐test, while normally distributed and heteroscedastic variables were compared using Welch's test. Non‐normally distributed variables were compared using the Mann–Whitney *U* test. Categorical variables are presented as numbers and percentages. For categorical data, groups were compared using the chi‐square test. Freedom from AT recurrence was analyzed using the Kaplan–Meier method with log‐rank *p* values. Statistical significance was defined as a two‐tailed *p* value < 0.05.

## 3 Results

**FIGURE 2:**
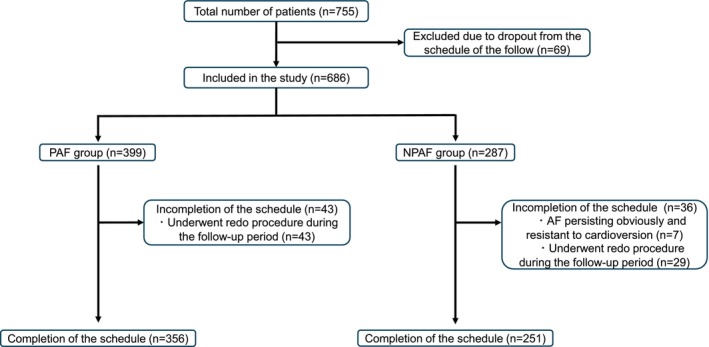
The study flow chart. A total of 69 patients dropped out, leaving 686 patients for analysis: 399 in the paroxysmal AF group and 287 in the non‐paroxysmal AF group. In the paroxysmal AF group, 43 patients discontinued the schedule due to redo procedures during follow‐up.

A total of 755 patients were included in the analysis. An overview of the study population is shown in Figure 2. Sixty‐nine patients were excluded because they dropped out during the follow‐up period. Of these, 50 patients were converted to 24‐h Holter monitoring during the follow‐up period as they declined the 2‐week Holter monitoring and preferred to continue with the 24‐h Holter monitoring, 15 patients dropped out of the hospital visit, and four patients died during the follow‐up period; two patients had malignancy, one patient had cerebral hemorrhage, and one patient had pneumonia. The remaining 686 patients were included in the study (399 and 287 in the PAF and NPAF groups, respectively). In the PAF group, 43 patients discontinued the schedule because they required repeated procedures during follow‐up. In the NPAF group, 36 patients discontinued: seven due to PEAF resistant to cardioversion and 29 due to repeated procedures. These patients who underwent a repeated procedure or were resistant to cardioversion were unable to complete the scheduled 2‐week Holter monitoring; however, the data from previously conducted 2‐week Holter monitoring were included in the analysis.

The baseline patients' characteristics are shown in Table 1. Body mass index, sleep apnea syndrome, congestive heart failure, hypertension, and diabetes mellitus were significantly higher in the NPAF group; the left ventricular ejection fraction was significantly lower, and BNP levels were higher. LA diameter, volume, and volume index were significantly larger in the NPAF group. CHADS2 and CHA2DS2‐Vasc scores were also higher in the non‐paroxysmal group. The rate of symptomatic AF (modified EHRA score ≥ 2a) [16] was significantly higher in the PAF group.

**TABLE 1:** Baseline patients' characteristics.

	ALL (*n* = 686)	PAF (*n* = 399)	NPAF (*n* = 287)	*p*
Age‐years	67.9 ± 10.1	68.3 ± 10.3	67.4 ± 9.9	0.26
Male sex, *n* (%)	463 (67.5%)	258 (64.7%)	205 (71.4%)	0.06
BMI	24.0 ± 3.8	23.5 ± 3.7	24.6 ± 3.9	< 0.001
SAS, *n* (%)	269/491 (54.8%)	128/260 (49.2%)	141/231 (61.0%)	< 0.01
CHF, *n* (%)	203 (29.6%)	67 (16.8%)	136 (47.4%)	< 0.001
HT, *n* (%)	432 (63.0%)	236 (59.1%)	196 (68.3%)	0.01
DM, *n* (%)	205 (29.9%)	105 (26.3%)	100 (34.8%)	0.02
Stroke/TIA, *n* (%)	55 (8.0%)	31 (7.8%)	24 (8.4%)	0.78
LVEF, %	62.3 ± 8.0	63.8 ± 7.2	60.2 ± 8.6	< 0.001
Cre, mg/dL	0.91 ± 0.34	0.90 ± 0.39	0.92 ± 0.25	0.28
HD, *n* (%)	11 (1.6%)	7 (1.8%)	4 (1.4%)	0.71
BNP, pg/mL	136.4 ± 167.1	101.2 ± 170.3	185.5 ± 149.7	< 0.001
LA diameter (echo), mm	38.4 ± 6.3	36.4 ± 5.6	41.4 ± 6.2	< 0.001
LA volume (CT), mL	123.8 ± 39.8	107.8 ± 31.6	146.2 ± 39.4	< 0.001
LAVI (CT), mL/m^2^	73.7 ± 23.6	65.3 ± 20.1	85.4 ± 23.2	< 0.001
CHADS2 score	1.7 ± 1.0	1.5 ± 1.2	1.9 ± 1.3	< 0.001
CHA2DS2Vasc score	2.7 ± 1.4	2.5 ± 1.6	2.9 ± 1.7	< 0.01
Symptom (modified EHRA score ≥ 2a), *n* (%)	577 (84.1%)	358 (89.7%)	219 (76.3%)	< 0.01

*Note:* Continuous variables are expressed as means and standard deviations. Categorical variables are presented as numbers and percentages.

Abbreviations: BMI, body mass index; BNP, brain natriuretic peptide; CHF, congestive heart failure; DM, diabetes mellitus; EHRA, European Heart Rhythm Association; HD, hemodialysis; HT, hypertension; LAVI, left atrial volume index; LVEF, left ventricular ejection fraction; SAS, sleep apnea syndrome; TIA, transient ischemic attack.

The ablation procedures are summarized in Table 2. Roof line and mitral isthmus line ablations were performed significantly more commonly in the NPAF group. Other procedures did not differ significantly between the two groups. There were four complications: one case of cardiac tamponade, one case of puncture site pseudoaneurysm, and two cases of transient decreased esophageal peristalsis.

**TABLE 2:** Ablation procedures.

	ALL (*n* = 686)	PAF (*n* = 399)	NPAF (*n* = 287)	*p*
PV isolation, *n* (%)	686 (100.0%)	399 (100.0%)	287 (100.0%)	—
PW isolation or touching rings, *n* (%)	302 (44.0%)	165 (41.4%)	137 (47.7%)	0.10
SVC isolation, *n* (%)	37 (5.4%)	22 (5.5%)	15 (5.2%)	0.87
Roof line, *n* (%)	4 (0.6%)	0 (0.0%)	4 (1.4%)	0.02
CTI ablation, *n* (%)	144 (21.0%)	85 (21.3%)	59 (20.6%)	0.81
MI ablation, *n* (%)	11 (1.6%)	2 (0.5%)	9 (3.1%)	< 0.01
Non‐PV foci ablation, *n* (%)	65 (9.5%)	36 (9.0%)	29 (10.1%)	0.63
Complication, *n* (%)	4 (0.6%)	1 (0.3%)	3 (1.0%)	0.18

*Note:* Categorical variables are presented as numbers and percentages.

Abbreviations: CTI, cavotricuspid isthmus; MI, mitral isthmus; PV, pulmonary vein; PW, posterior wall; SVC, superior vena cava.

**FIGURE 3:**
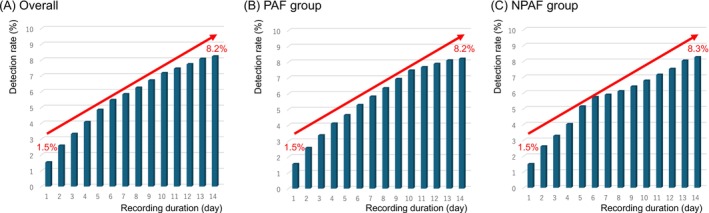
The cumulative detection rate of any atrial tachyarrhythmia in 2‐week Holter monitoring after AF ablation increased gradually with each passing day in all groups. The cumulative detection rate increased approximately 5.5 times in 14 days compared to 1 day. (A) Overall. (B) PAF group. (C) NPAF group.

The cumulative detection rates of any AT recorded during the 2‐week Holter monitoring period following AF ablation are shown in Figure 3. The cumulative detection rate increased steadily from 1.5% on day 1 to 8.2% on day 14; similar results were observed when the data were analyzed separately for the PAF and NPAF groups. The recurrences within the first 24 h were detected in 31 records in the PAF group and in 20 records in the NPAF group. During the remaining 13 days of the observation period, multiple episodes of recurrence were observed in 20 records (64.5%) and persistent AF was observed in 1 record (3.2%) in the PAF group. In the NPAF group, multiple recurrences were observed in 4 records (20.0%), and persistent AF was observed in 14 records (70.0%). The median monitoring duration was 13.6 ± 1.1 days. Approximately 84% of patients wore the device for 14 days, and approximately 97% of patients wore the device for 10 days or more. Among the patients with recurrence, the number of patients who presented with arrhythmia symptoms but were not detected by the monitoring device was eight (7.8%) in the PAF group and four (5.7%) in the NPAF group.

**FIGURE 4:**
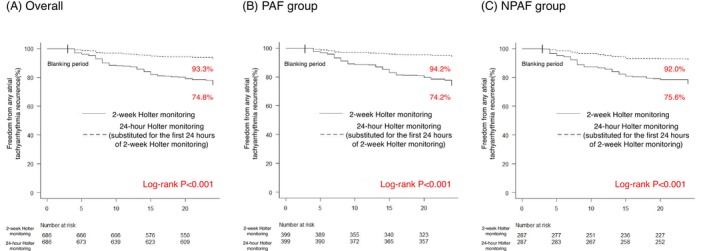
Kaplan–Meier curves showed that in all groups, the rate of freedom from any atrial tachyarrhythmia recurrence was significantly lower with 2‐week Holter monitoring follow‐up than with 24‐h Holter monitoring follow‐up. It means that 2‐week Holter monitoring significantly outperforms 24‐h Holter monitoring in detecting atrial tachyarrhythmia recurrence. (A) Overall. (B) PAF group. (C) NPAF group.

The clinical outcome and Kaplan–Meier curves of freedom from AT recurrence after AF ablation are shown in Figure 4. During the 2‐year follow‐up period, the overall success rate was 74.8% when followed by a 2‐week Holter monitoring. If followed by 24‐h Holter monitoring, the success rate was 93.3%, and similar results were observed when the data were analyzed separately between the PAF and NPAF groups.

**FIGURE 5:**
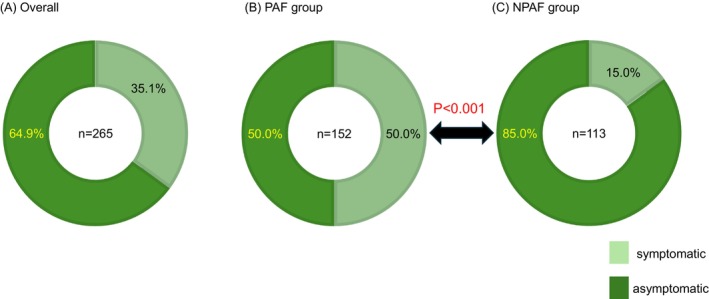
The rate of asymptomatic recurrence in Holter records was 64.9% overall, 50.0% in the PAF group, and 85.0% in the NPAF group. In the NPAF group, most of the recurrence form was asymptomatic. (A) Overall. (B) PAF group. (C) NPAF group.

The rates of symptomatic and asymptomatic recurrence in the Holter recordings are shown in Figure 5. Approximately 65% of the recurrences were asymptomatic; the asymptomatic recurrence rate was significantly more frequent in the NPAF group (85.0%) than in the PAF group (50.0%) (*p* < 0.001). The rates of symptomatic and asymptomatic AF before the procedure were 577 patients (84.1%) and 109 patients (16.9%), overall, respectively: 358 (89.7%) and 41 (10.3%) in the PAF group, and 219 (76.3%) and 68 (23.7%) in the NPAF group, respectively. After the procedure, the rates of symptomatic and asymptomatic AF recurrence were 62 patients (35.8%) and 111 patients (64.2%), overall, respectively: 50 (48.5%) and 53 (51.5%) in the PAF group, and 12 (17.1%) and 58 (82.9%) in the NPAF group, respectively. Among 25 patients with recurrence who had asymptomatic AF before the procedure (8 in the PAF and 17 in the NPAF group), 22 patients (88.0%) experienced asymptomatic recurrence (5 in the PAF and 17 in the NPAF group). Among 148 patients with recurrence who had symptomatic AF before the procedure (95 in the PAF and 53 in the NPAF group), 89 (60.1%) experienced asymptomatic recurrence (48 in the PAF and 41 in the NPAF group).

Overall, 238 patients (144 in the PAF group and 94 in the NPAF group) had a CHADS2 score of 0 or 1, and 194 patients (81.5%) (125 in the PAF group and 69 in the NPAF group) discontinued anticoagulation therapy. The remaining patients who continued anticoagulation therapy included those with a history of percutaneous coronary intervention or valvular disease surgery and those who requested continuation.

## 4 Discussion

The main findings of the present study are as follows: (1) 2‐week Holter monitoring could significantly detect the recurrence of any AT after CA compared to 24‐h Holter monitoring. (2) Asymptomatic recurrence was significantly higher in the NPAF group than in the PAF group.

Twenty‐four‐hour Holter monitoring is commonly used to evaluate long‐term outcomes after CA [17, 18], owing to its simplicity compared to long‐term Holter monitoring. However, the Kaplan–Meier curves in this study demonstrated that approximately 20% of recurrence may have been missed during the 2‐year follow‐up period with 24‐h Holter monitoring compared to 2‐week Holter monitoring. Recent reports indicate that long‐term Holter monitoring is often preferred for follow‐up after CA [12, 13], and it plays a critical role in determining whether symptoms such as palpitations are attributable to recurrence. Previous reports have shown that PACs or premature ventricular contractions frequently cause palpitations, which are unreliable predictors of recurrence [19, 20].

In a previous report, the detection rate of AF using 30‐day Holter monitoring generally peaked at 2 weeks and leveled off thereafter [21]. Many wearable and portable devices are now available for AF detection, and the utility of smartwatches for this purpose has been reported [22]. Several clinical studies have reported that implantable cardiac monitoring (ICM) has a higher detection rate of AF than conventional monitoring in patients with stroke [21, 23] and those who have undergone AF CA [24, 25]. ICM has shown promise as a long‐term follow‐up device for patients after CA; however, it is invasive and has several limitations: it may under‐sense the beats and over‐sense the electromyographic signals, or overdiagnose PACs and PVC as AF, and it is not recommended in the current guidelines [26]. Because few countries have insurance coverage for ICM as a follow‐up device after CA, the use of a wearable device is appropriate. An expert consensus statement suggests that in the absence of invasive monitoring, the intermittent use of Holter monitoring, especially longer‐duration monitoring, is preferable as a follow‐up device after CA [10].

The device used in this study was a thin, lightweight (25 g), waterproof polyurethane patch that allowed patients to shower or take partial baths while wearing, causing minimal burden. Patch‐type devices offer several advantages: they are thin, compact, user‐friendly, and enable long‐term monitoring with minimal patient burden, improving the AF detection [27]. However, their disadvantage is the single‐lead recording, which can make arrhythmia diagnosis challenging when the P‐wave amplitude is low [28, 29].

Previous studies reported that the ratio of asymptomatic to symptomatic AF episodes increases after CA. Verma et al. reported that the ratio of asymptomatic to symptomatic AF episodes increased from 1.1 before CA to 3.7 after CA [11]. In other reports, the rate of asymptomatic AF increased from 11% to 35% before CA and from 53% to 65% after CA [30, 31, 32]. In this study, 64% of overall recurrences were asymptomatic, with a particularly high rate of 85% in the NPAF group. In general, patients with PAF are more likely to experience palpitations, whereas those with NPAF tend to experience symptoms such as dyspnea or fatigue [33, 34]. During its natural time course, AF commonly progresses from asymptomatic and undiagnosed PAF to persistent or permanent AF [35, 36]. Patients with NPAF are often asymptomatic during the paroxysmal phase and typically present to the hospital with complaints of dyspnea or fatigue, which develop as the arrhythmia progresses to a persistent phase, often without patient awareness. The difference, whether symptomatic or asymptomatic, at the time of recurrence after CA may be attributed to the nature of symptom presentation in each type of AF. Patients with PAF frequently experience palpitations from onset and are more likely to experience their recurrence. Patients with NPAF are often asymptomatic during the paroxysmal phase and may remain unaware of recurrence until more symptoms develop. Because of this, the assessment of recurrence based on 24‐h Holter monitoring and/or subjective symptoms may underestimate recurrence after AF ablation. Long‐term Holter monitoring, such as that for 2 weeks, may be preferred for follow‐up after CA in patients with AF.

Intermittent ECG monitoring after CA may help determine whether anticoagulation therapy should be continued after the procedure. Current guidelines address whether to continue anticoagulation after CA: the Japanese Circulation Society (JCS) guidelines recommend that it be continued if the CHADS2 score is ≥ 2 [37], the American Heart Association guidelines recommend that it be continued if the CHA2DS2Vasc score is ≥ 2 [9]; the European Society of Cardiology guidelines recommend that it should be based on the patient's stroke risk and preference [36]. In this study, anticoagulation was discontinued 3–6 months after CA in patients without recurrence, according to the JCS guidelines. To date, no large randomized controlled trials have been conducted to evaluate the safety of discontinuing anticoagulation therapy after CA; however, one recent report indicated that continued anticoagulation therapy after CA resulted in a higher risk of major bleeding in patients with a CHADS2 score < 2 and a lower risk of thromboembolism in patients with a CHADS2 score ≥ 3 [38]. A recent study evaluated thromboembolic events, major bleeding events, and all‐cause mortality in patients without recurrence at 12 months after CA, stratified into OAC continuation and discontinuation groups [39]. In this study, asymptomatic AF, left ventricular ejection fraction < 60%, and LA diameter > 45 mm were identified as risk factors for thromboembolic events. Although follow‐up was conducted using 24‐h Holter ECG monitoring, the authors noted that the limited duration and frequency of monitoring, as well as the low probability of detecting intermittent AF, likely contributed to the under‐recognition of AF recurrence and thromboembolic events. These findings underscore the importance of improving AF detection rates by extending the monitoring period, such as through the repeated use of long‐term Holter monitoring, including 2‐week Holter monitoring. One of the advantages of using 2‐week Holter monitoring for the evaluation of recurrence after CA is that it helps clinicians make informed decisions about continuing anticoagulation therapy, allowing it to be discontinued relatively safely owing to more accurate detection of recurrence. In this study, none of the patients who discontinued anticoagulation therapy experienced thromboembolic events.

## 5 Study Limitation

This study had several limitations. First, this was a non‐randomized study. Twenty‐four‐hour Holter monitoring was not performed and was substituted for the first 24 h of 2‐week Holter monitoring. Therefore, if atrial tachyarrhythmia recurrence was confirmed by 2‐week Holter monitoring and the patients subsequently underwent a repeated procedure, but no recurrence was detected in 24‐h Holter monitoring, the patients were considered to have no event at that time and excluded from the subsequent Kaplan–Meier analysis. Of the 72 patients who underwent repeated procedure, 42 (58.3%) were eligible; however, these patients did not complete the scheduled 2‐week Holter monitoring. Consequently, the recurrence rate in the 24‐h Holter monitoring group may have been underestimated. This limitation could be addressed in future studies through the conduct of randomized controlled trials. Second, this was a retrospective and observational study. Third, this study was performed at a single center with a small sample size. Fourth, not all patients could wear the device for 2 weeks; 84.1% of the patients completed 14 days of 2‐week Holter monitoring (83.0% in the PAF group and 84.9% in the NPAF group); however, 97% of the patients wore the device for 10 days or more. Finally, additional ablation was performed at the discretion of the operator, which may have influenced the outcomes. Further prospective and randomized trials with large sample sizes are necessary to confirm the results of this study.

## 6 Conclusions

Two‐week Holter monitoring was more effective than conventional 24‐h Holter monitoring in detecting AT recurrence after AF CA in patients with AF. Especially in the NPAF group, 2‐week Holter monitoring was valuable for follow‐up after AF CA owing to the high rate of asymptomatic recurrence. The findings indicate that 2‐week Holter monitoring may be effective for a more accurate assessment of recurrence and decision to continue anticoagulation therapy after AF CA.

## Ethics Statement

This study was approved by the Institutional Review Board of Toyohashi Heart Center (250201). This study was conducted in accordance with the ethical principles outlined in the Declaration of Helsinki. All authors confirm that the research adheres to the ethical guidelines of the Journal of Arrhythmia and follows the established standards of research integrity, including proper citation and avoidance of plagiarism.

## Consent

Informed consent was obtained from all participants.

## Conflicts of Interest

The authors declare no conflicts of interest.

## Data Availability

The datasets that support the findings of the current study are available from the corresponding author, upon reasonable request.
